# The birth of the European Liver transplant registry

**DOI:** 10.3389/ti.2026.17721

**Published:** 2026-10-22

**Authors:** Henri Bismuth

**Affiliations:** Centre Hépatobiliaire, Hôpital Paul Brousse, Villejuif, France

**Keywords:** consensus conference, European liver transplant registry, hepatitis B, history of medicine, liver transplantation

## Abstract

This personal account, written on the occasion of the fortieth anniversary of the European Liver Transplant Registry (ELTR), traces the origins of the Registry from the earliest days of liver transplantation in Europe to its present scale. Beginning with a handful of pioneering centres in the 1970s and the scepticism that surrounded the procedure, the narrative follows the events of 1983 that transformed the field, the creation of an informal registry at Paul Brousse, and its extension to a European network following a founding meeting in Hannover in October 1985. It describes the early design choices that shaped the Registry, the methodological debates that defined its core definitions, and the scientific contributions that followed, including landmark findings on patient survival and on hepatitis B recurrence after transplantation, as well as the 1993 Paris Consensus Conference on indications for liver transplantation. The account closes with the Registry’s transition to new stewardship and its present scope, spanning more than 200,000 transplants recorded across 32 countries. The history illustrates how informal, trust-based collaboration among a small number of European surgeons gave rise to one of the longest-running transplant registries in the world.

## Introduction

This manuscript, which I am honoured to present on the occasion of the European Liver Transplant Registry’s (ELTR) fortieth anniversary, tells the story of how it began, and of the European transplant community that made it possible.

## Origins and early context

There is, as with any birth, a precise date - 16 October 1986 - and a precise place: Hannover. And before this “birth” came its “pregnancy”: a year earlier in Paris, when the small group of French surgeons beginning liver transplantation met to share their first experiences.

I recall those years vividly. When we performed our first liver transplant at Paul Brousse in 1974, a single case (that, at the time, represented twenty-five per cent of all European experience!), I could not have imagined where that first operation would lead. Liver transplantation in Europe was confined to a very small number of centres. Cambridge, under Roy Calne, and Hannover, under Rudolph Pichlmayr, were the dominant units. A handful of others, Vienna, Paul Brousse, Innsbruck, and Groningen, had performed only modest numbers of procedures, and some experience was frankly anecdotal (Table 1).

**TABLE 1:** European Canters’ activity before 1984 (17 centers).

Center	Head	Date of 1st LT	No LT before 1984
Cambridge	R. Calne	02/05/1968	147
Wien	F. Pisa	10/07/1972	17
Hannover	R. Pichlmayr	25/11/1972	109
Paris P. Brousse	H. Bismuth	14/01/1974	17
Bordeaux	J. Saric	06/09/1974	2
Innsbruck	R. Margreiter	03/02/1977	11
Strasbourg	J. Cinqualbre	02/02/1978	6
Rennes	B. Launois	21/04/1978	6
Groningen	MJH. Sloof	12/03/1979	34
Birmingham	P. McMaster	23/01/1982	9
Paris Cochin	Y. Chapuis	29/03/1982	1
Roma	R. Cortesini	20/05/1982	2
Helsinki	C. Hockerstedt	12/12/1982	1
Berlin	R. Häring	12/04/1983	2
Brussels	JB. Otte	21/12/1983	1
Milan	D. Galmarini	09/06/1983	1
Bern	P. Gertsch	19/12/1983	2

The clinical atmosphere was far from encouraging. Patients were referred for transplantation only as a last resort, and mortality was substantial. Dame Sheila Sherlock of the Royal Free Hospital had written that a liver transplant recipient could be regarded as fortunate if he survived 6 months post-operatively, and that the procedure was not justified when the natural course of disease would allow the patient to live longer. In July 1980, Thomas Starzl published data documenting a decline in survival after liver transplantation, reinforcing the prevailing scepticism [1].

## The 1983 turning point

Two events in 1983 changed the landscape decisively. First, the Washington Consensus Development Conference addressed the question of whether liver transplantation constituted a genuine treatment for end-stage liver disease. At that time, the US insurance companies routinely declined to reimburse the procedure because it remained experimental. Following deliberations led principally by Thomas Starzl, the Conference concluded that liver transplantation was a promising alternative to current therapy in the management of the late phase of several forms of serious liver disease, and that it had the potential to transition from an experimental procedure to a clinical service [2]. This endorsement was pivotal.

Second, the clinical introduction of cyclosporine, through the pioneering work of Roy Calne, transformed immunosuppressive management and dramatically improved outcomes. Together, these developments produced a striking inflection in activity: annual liver transplant volumes rose from single figures to two-digit numbers within a very short period (Figure 1).

**FIGURE 1:**
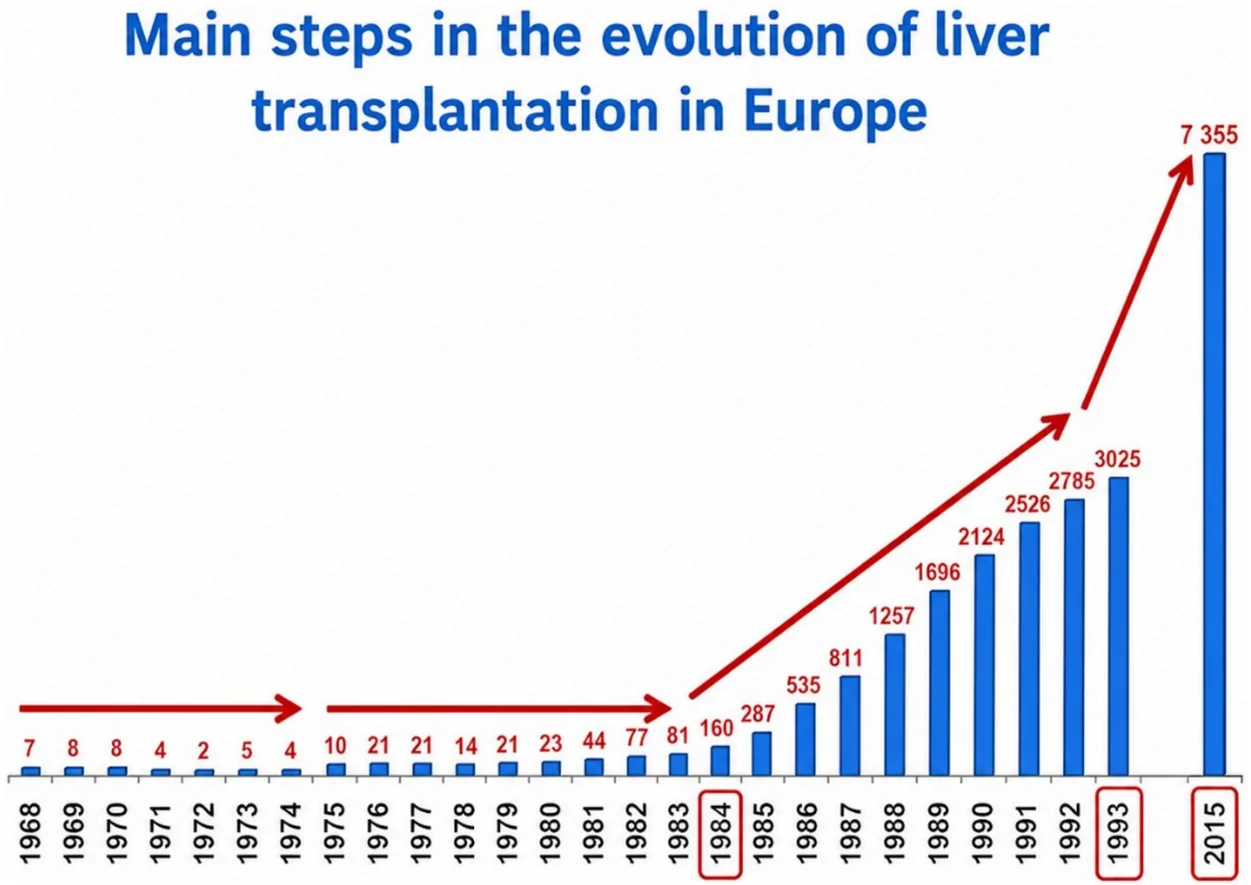
Annual liver transplant volume in Europe, 1968–2015.

## The French prototype registry

In 1984, the Paul Brousse experience was still very limited: only 19 cases over 10 years. Other French centres soon joined us - Strasbourg, Rennes, Bordeaux, Cochin, and Saint-Antoine. We were working in relative isolation within our hospitals; most hepatologists showed little interest, and the literature was scarce and largely experimental. We therefore decided to hold regular meetings to discuss our patients. To prepare for these meetings and structure our discussions, I proposed to compile our cases in advance in a sort of registry, which was circulated to the group before each meeting. It was a simple list of cases, recording disease aetiology, type of transplant, and key complications. This informal French registry, born at Paul Brousse, served as the direct model for what would follow at the European level.

When the European Society for Organ Transplantation (ESOT) held its inaugural congress in 1983 in Zurich, there were only two presentations on liver transplantation. But for the first time, the small group of European liver transplant surgeons met. At the subsequent International Transplantation Society congress, in 1984, our group decided to convene a dedicated liver transplantation session at the second ESOT congress, scheduled for late November 1985 in Munich, with the session organised by Rudolf Pichlmayr (Figure 2).

**FIGURE 2:**
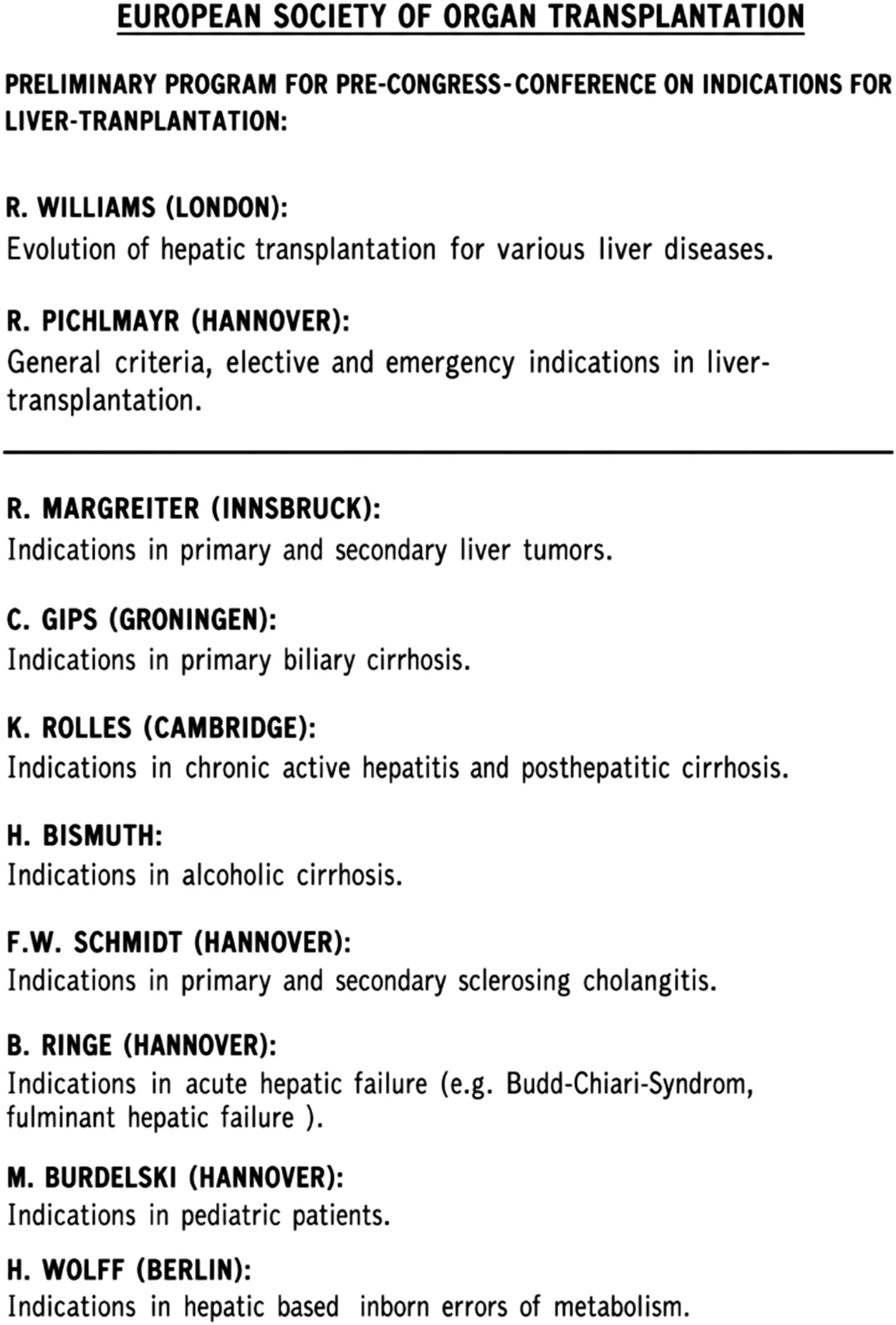
Pre-congress programme, second ESOT Congress, November 1985.

## The founding meeting, hannover, 16 October 1985

One month before the Munich congress, Pichlmayr convened a preparatory meeting in Hannover on 16 October 1985. Those present included Roy Calne, Carl Groth, Maarten Sloof, and myself, five participants in all. It was at that meeting that I proposed extending the French registry to the European level. The proposal was immediately accepted, and I confess that the swiftness of that acceptance surprised even me.

At the subsequent Munich ESOT on 27 November 1985 (Figure 3), at the end of the Liver session, I proposed the concept of this Registry.

**FIGURE 3:**
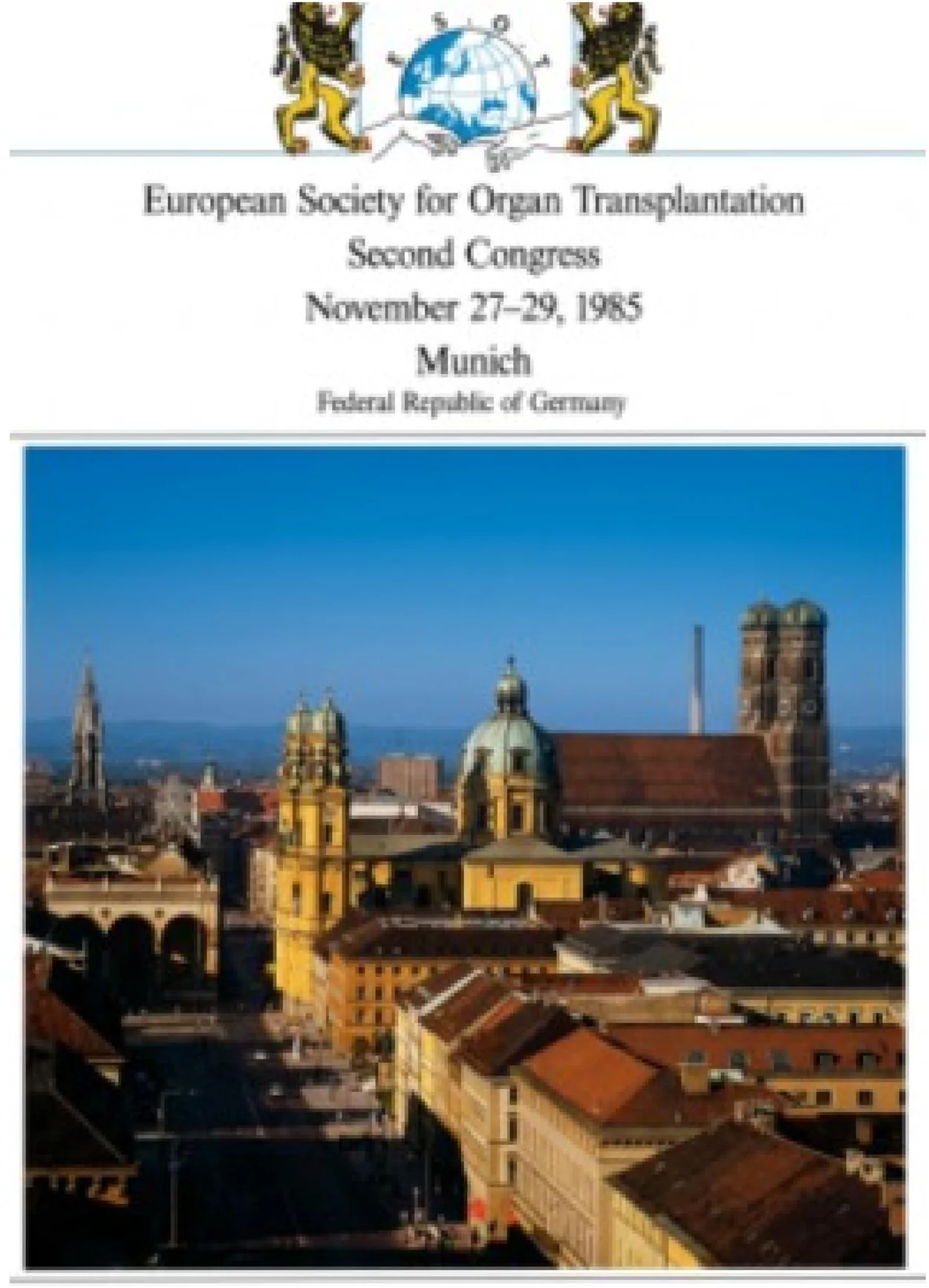
Second ESOT Congress, Munich, November 27–29, 1985.

A short circular was distributed, the first document to bear the name “European Liver Transplant Registry” (Figure 4), inviting centers to participate in what we described as the first collaborative exchange of the European liver grafting groups. The response was positive.

**FIGURE 4:**
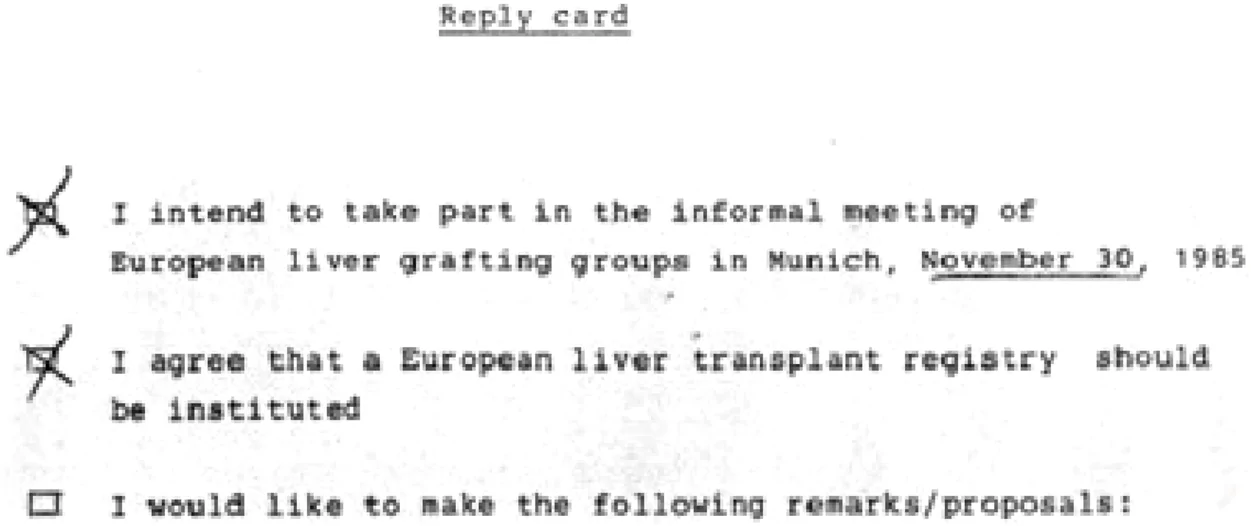
Reply card circulated to potential ELTR participants, November 1985.

A coordinating committee was constituted, comprising Bo-Göran Ericzon, Jean-Bernard Otte, Burckhardt Ringe, Keith Rolles, and Maarten Sloof. The first formal ELTR meeting was held in Paris on 7 January 1986, at which the registry’s objectives were defined (Figure 5).

**FIGURE 5:**
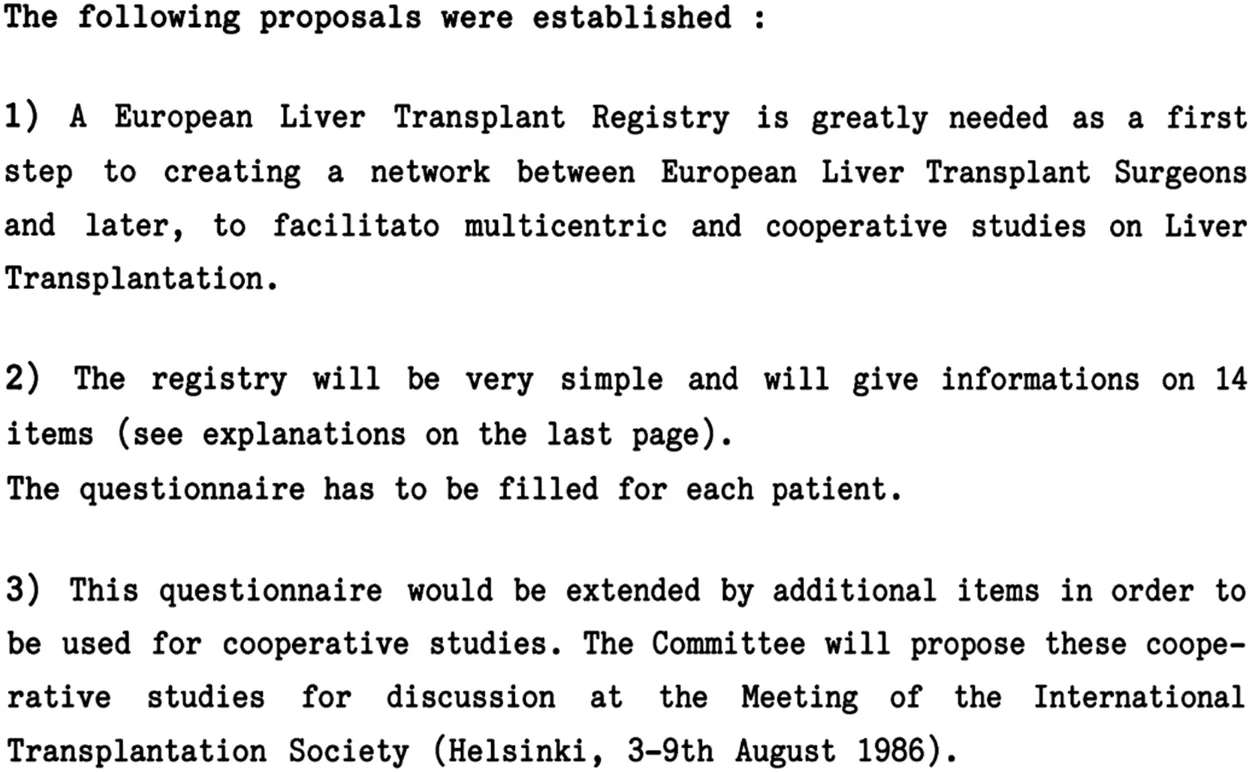
Founding objectives of the ELTR (1986).

## Design principles and early operation

From the outset, I insisted that the ELTR questionnaire be kept deliberately simple (Figure 6). Simplicity was, in my view, essential for ensuring compliance from contributing centres. Coverage was intended to be comprehensive: all liver transplants performed in Europe were to be included, so as to produce a representative and statistically robust dataset. With Cambridge and Hannover, the two largest units, having agreed to participate, we assumed that others would follow, and they did.

**FIGURE 6:**
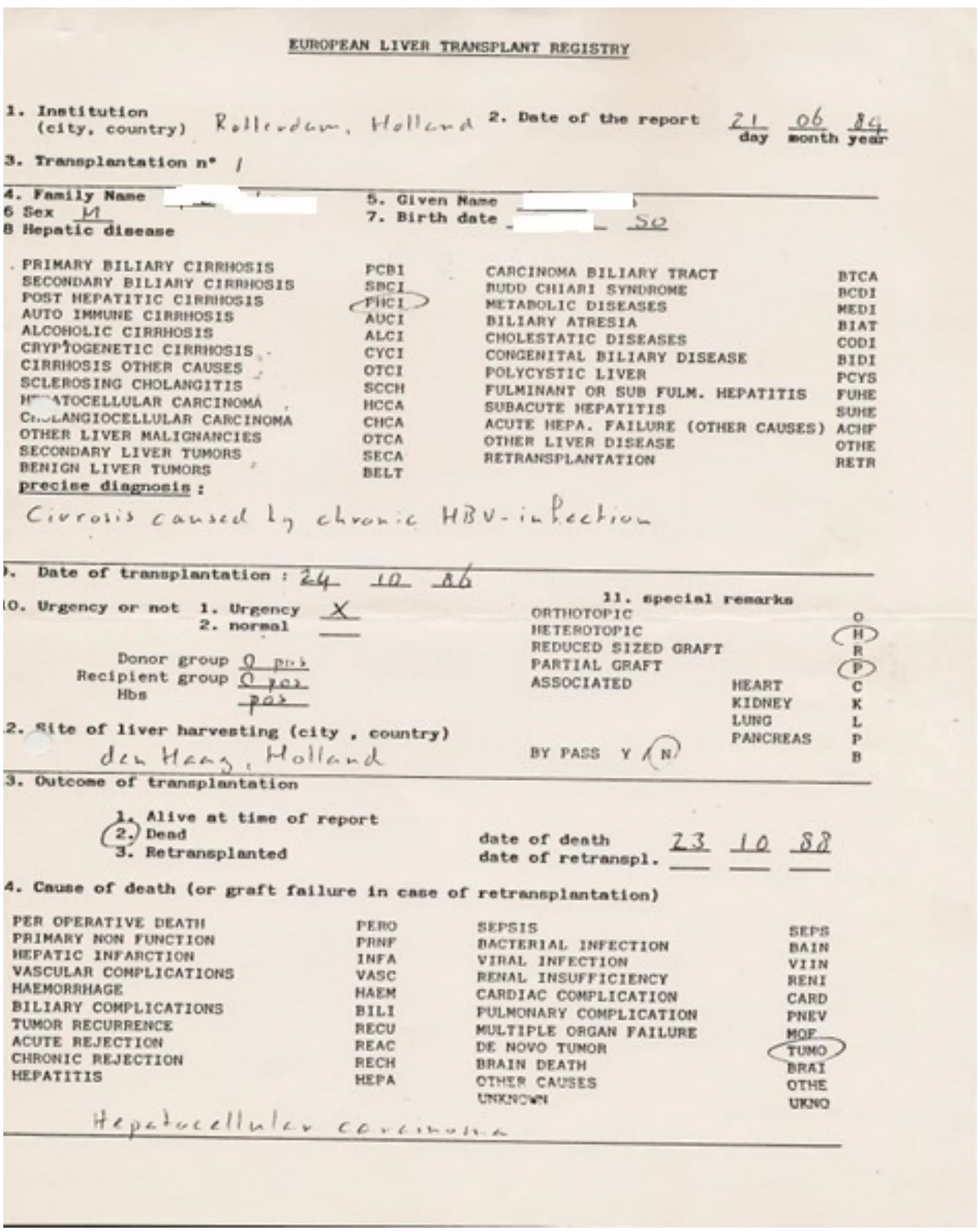
First ELTR questionnaire (1986 - 21 items).

Updates were planned on a quarterly basis, an ambitious schedule that proved workable in practice. Because neither personal computers nor the internet existed in any practical form at the time, data submission relied entirely on paper questionnaires. Hundreds of completed forms were received at Paul Brousse. I must pay particular tribute to my co-worker Denis Castaing, whose management of data entry and statistical analysis was indispensable to the early operation of the registry. Without his rigour, the ELTR could not have functioned.

It was only on 15 September 1995 that Vincent Karam joined the ELTR team, initiating the shift towards a fully professionalised secretariat, with biostatistician Valérie Cailliez and secretary Eva Cochet, whose work would underpin and advance the Registry in the years that followed.

## Methodological challenges

Early operation revealed important definitional ambiguities. When Paul Brousse submitted a case of intraoperative death due to haemorrhage, it appeared to be the first case reported, and it became apparent that other centres had excluded such events because transplantation had not been completed. This discrepancy prompted a formal debate on when transplantation should be considered to have occurred and should be registered: at anaesthetic induction, at laparotomy, at hepatectomy, at graft implantation, or at vascular declamping?

We reached consensus that transplantation is complete, and the patient is therefore registrable, at the moment of vascular declamping, when the graft first becomes functional. That definition has remained in use ever since, though I have always acknowledged its imperfections: in hepatic resection, deaths at any point during the procedure are attributed to the operation, a standard that our transplant definition does not fully replicate.

## Scientific contributions

Data collection began in 1986, and the first ELTR report was published the following year in The Lancet, in September 1987, based on 1,218 patients [3]. It was, at the time, the largest liver transplant series in the world, assembling data from centres across Europe. One-year and two-year patient survival rates of 44% and 41%, respectively, constituted a definitive rebuttal of the earlier pessimism that had surrounded the field, and confirmed liver transplantation as a valid therapeutic option for end-stage liver disease (Figure 7).

**FIGURE 7:**
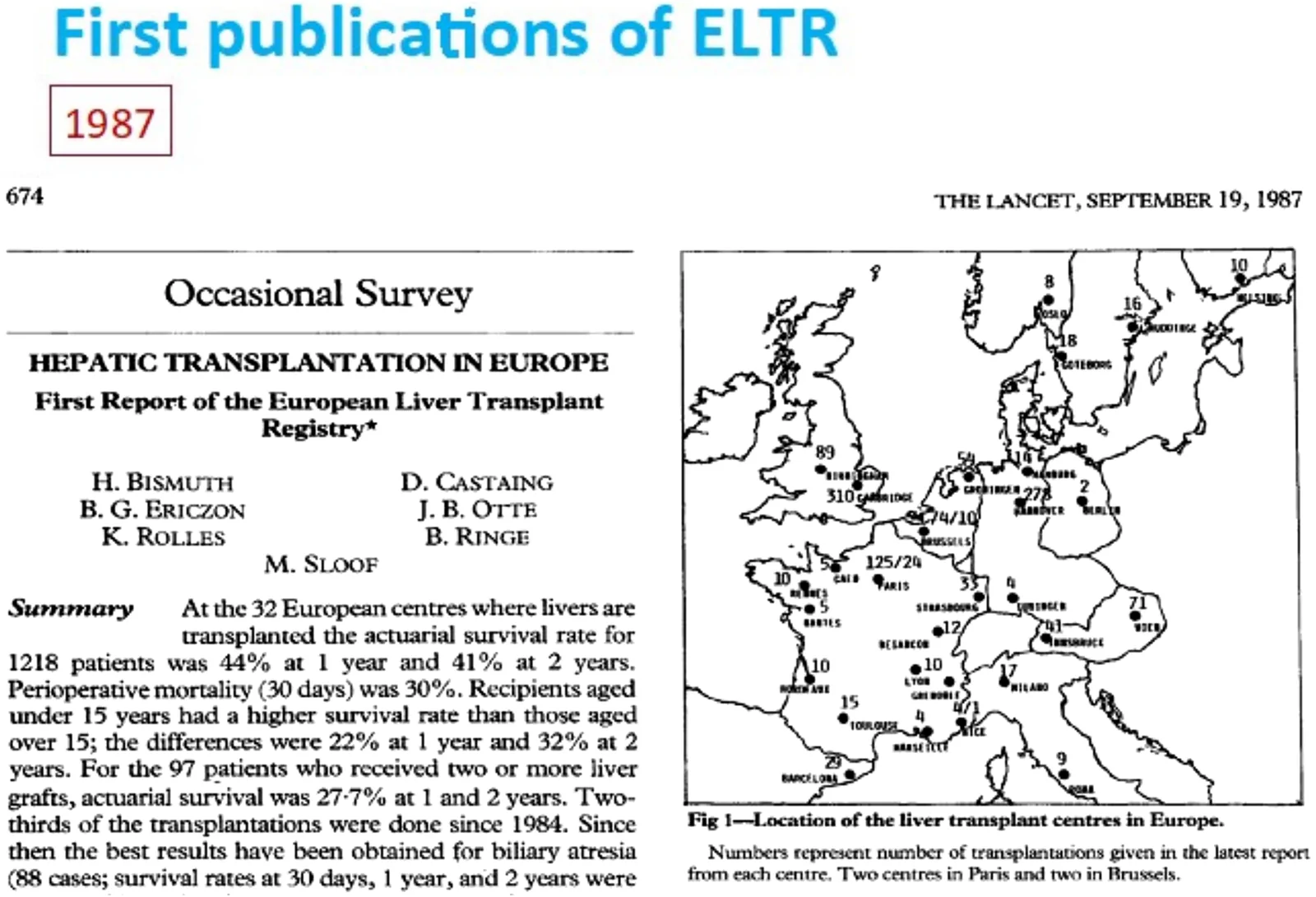
First ELTR publication (The Lancet, 1987).

The Registry had been criticised since the beginning for the paucity of the data collected, not enabling it to be used for valuable scientific studies. The registry’s second major scientific contribution arose from work on hepatitis B virus (HBV) recurrence after transplantation. My colleague Didier Samuel was investigating prophylactic immunoglobulin therapy to prevent HBV recurrence on the graft, but individual centres had insufficient patient numbers for a convincing study. However, the Registry allowed us to identify the centres with the largest number of HBV-transplanted patients through the ELTR network. By distributing to these centers an extended questionnaire, we assembled a collaborative dataset that formed the basis for a landmark publication in the New England Journal of Medicine in 1993 [4]. That paper established prophylactic immunoglobulin as the standard of care for HBV-infected transplant recipients, and I regard it as one of the registry’s most consequential contributions.

A further landmark came in June 1993 with the second Consensus Development Conference, held in Paris exactly 10 years after the Washington conference. That Conference aimed to answer the question: Is Liver Transplantation a therapeutic procedure? Now the question was: To which patient? Drawing on the ELTR database, an international panel that included Thomas Starzl, Ronald Busuttil, Nancy Ascher, and European liver transplant surgeons and hepatologists (Roy Calne, Roger Williams, Rudolf Pichlmaier, Carl Groth, Bo-Göran Ericzon, Jean-Pierre Benhamou, Marteen Sloof, Jean Bernard Otte, Paul McMaster) produced a consensus statement defining the actual indications for liver transplantation. The Paris Consensus, published in a special issue of Hepatology, shaped clinical practice globally for years thereafter [5].

## Transition and maturity

In 1997, twelve years after the registry’s foundation, I transferred stewardship of the ELTR to René Adam, who guided the Registry through its consolidation and expansion.

Quality assurance became an explicit priority, with audit visits to contributing centres initiated in 1998 and led by Vincent Karam, who assembled and coordinated a multinational team to verify the accuracy and completeness of the data.

A formal quality-control programme, reported in 2003, confirmed the registry’s reliability and credibility as a representative source of data on European liver transplant practice [6].

The ELTR predated the European Liver and Intestine Transplant Association (ELITA) and may have contributed to the conditions that enabled its formation. Equally, the registry’s pan-European scope gave it a significance beyond the purely scientific: at its inception, European scientific cooperation was still in its infancy, and the ELTR offered a model for collaborative data-sharing that transcended national boundaries.

## The ELTR today

Today, the ELTR has accumulated data on more than 200,000 liver transplants performed in 174 centres across 32 countries. More than 114 peer-reviewed publications have drawn on its dataset, and registry data have underpinned the development of risk models for predicting liver transplant outcomes. The ELTR owes its enduring success not only to its founders, successive custodians, and ELITA board members, but also to the sustained commitment of all contributing European liver transplant centres and organ-sharing organisations (Figure 8).

**FIGURE 8:**
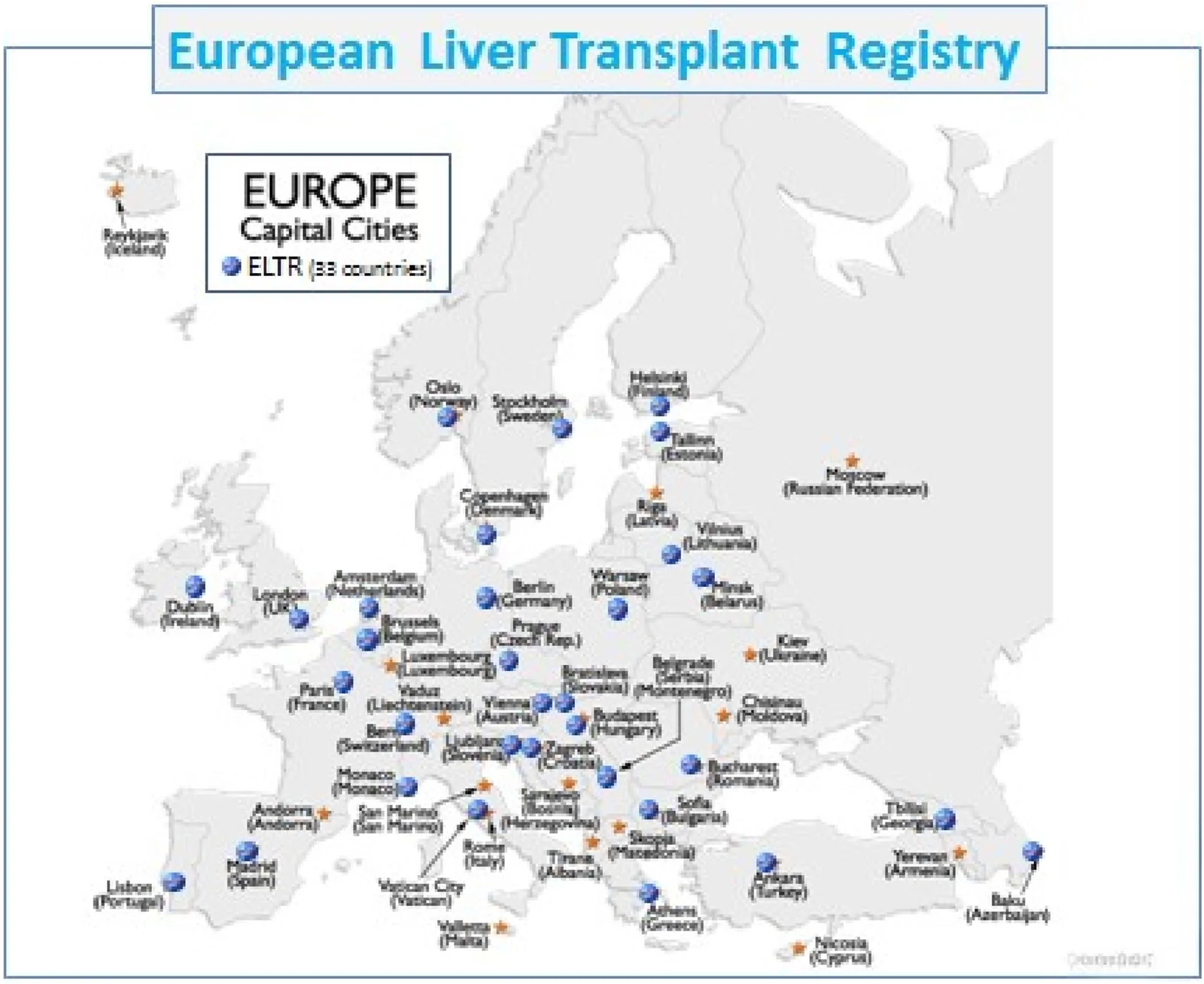
European Liver Transplant Registry - 32 participating countries.

Looking back, what strikes me most is not the scale the registry has reached, but the simplicity of what began it: a handful of surgeons, in Hannover on an October afternoon, agreeing that we would do better together than apart. The ELTR was built on trust, and it is trust that has sustained it.

## Data Availability

The original contributions presented in the study are included in the article/supplementary material, further inquiries can be directed to the corresponding author.
